# Patient-reported outcome measures (PROMs) after elective hip, knee and shoulder arthroplasty: protocol for a prospective cohort study

**DOI:** 10.1186/s12891-019-2745-3

**Published:** 2019-08-15

**Authors:** Alberto Grassi, Davide Golinelli, Dario Tedesco, Maurizia Rolli, Barbara Bordini, Marilina Amabile, Paola Rucci, Maria Pia Fantini, Stefano Zaffagnini

**Affiliations:** 1 IIa Clinica Ortopedica e Traumatologica, IRCCS Istituto Ortopedico Rizzoli, Bologna, Italy; 2 Department of Biomedical and Neuromotor Sciences, University of Bologna, Bologna, Italy; 3 IRCCS Istituto Ortopedico Rizzoli, Bologna, Italy; 4 Medical Technology Lab, IRCCS Istituto Ortopedico Rizzoli, Bologna, Italy

**Keywords:** Arthroplasty, Patient reported outcome measures, Implant registry

## Abstract

**Background:**

The number of hip, knee and shoulder arthroplasties continues to rise worldwide. The Organization for Economic Cooperation and Development has launched an initiative (called PaRIS Initiative) for the systematic collection of Patient Reported Outcome Measures (PROMs) in patients undergoing elective hip and knee arthroplasty. The Rizzoli Orthopedic Institute (IOR) was selected as a pilot center for the launch of the Initiative in Italy given that IOR hosts the Registry of Orthopedic Prosthetic Implants (RIPO), a region-wide registry which collects joint implant data from all the hospitals in the Emilia-Romagna Region. In this specific geographic area information related to PROMs after joint replacement is unknown. This paper describes the protocol of a study (PaRIS-IOR) that aims to implement the collection of a set of PROMs within an existing implant registry in Italy. The study will also investigate the temporal trend of PROMs in relation to the type of prosthesis and the type of surgical intervention.

**Methods:**

The PaRIS-IOR study is a prospective, single site, cohort study that consists of the administration of PROMs questionnaires to patients on the list for elective arthroplasty. The questionnaires will be administered to the study population within 30 days before surgery, and then at 6 and 12 months following surgery. The study population will consist of consecutive adult patients undergoing either hip, knee or shoulder arthroplasty. The collected data will be linked with those routinely collected by the RIPO in order to assess the temporal trend of PROMs in relation to the type of prosthesis and the type of surgical intervention.

**Discussion:**

The PaRIS-IOR study could have important implications in targeting the factors influencing functional outcomes and quality of life reported by patients after hip, knee and shoulder arthroplasty, and will also represent the first systematic collection of PROMs related to arthroplasty in Italy.

**Trial registration:**

Protocol version (1.0) and trial registration data are available on the platform www.clinicaltrial.gov with the identifier NCT03790267, first posted on December 31, 2018.

## Background

The number of hip, knee and shoulder arthroplasties continues to rise worldwide [1]. Arthroplasties are increasing due to several factors, such as the higher longevity and higher osteoarthritis incidence [2–4]. This represents a challenge for healthcare providers, given its consequences on patients’ functional outcomes and quality of life, and related costs.

The World Health Organization (WHO) has defined patient centeredness as a fundamental characteristic for the quality of healthcare [5]. Patient centeredness can be improved through the collection and analysis of functional outcomes and quality of life reported by patients, the so-called Patient-Reported Outcome Measures (PROMs). PROMs take the perspective of the patient, and typically represent outcomes that matter more to patients, such as impact on usual activities and self-care, and are described in a language that is easier to understand than clinical measures [1]. Measuring PROMs in patients who have undergone arthroplasty surgery can be a key tool to improve healthcare quality. PROMs are routinely collected in the implant registries of several OECD countries [6, 7]. For example, the National Joint Registry (NJR) in the United Kingdom has one of the largest PROMs collections of patients undergoing hip, knee or shoulder arthroplasty [8]. Studies from Sweden, Norway, and the Netherlands showed that adding the patient perspective by integrating data on functional outcomes and quality of life to selected health information systems, such as registries, gives relevant benefits in terms of quality improvement [9–11]. However, there are differences in the quality of life in such different countries. One important consideration when using such measures in multi-country studies is that many geographical factors (ethnicity, culture, etc.) may potentially affect responses in patient reported outcomes. This can make the evidence originated from a country registry (or a PROMs collection) non-generalizable. In fact, multiple studies comparing various national arthroplasty registries highlighted difference in pre-operative patients’ characteristics and PROMs [12–16], thus cautioning from within-countries comparison and suggesting risk-adjustment prior to making conclusions about apparent differences in outcome.

Moreover, PROMs assessment is not extensively adopted in many contexts due to the difficulty in systematically recording information reported by patients before and after the surgical procedure. A standard of PROMs collection is therefore necessary in as many countries as possible. Accordingly, the Organization for Economic Cooperation and Development (OECD) has launched an initiative (Patient-Reported Indicator Survey, PaRIS) for the systematic collection of PROMs in patients undergoing elective hip and knee prosthetic arthroplasty at 6 and 12 months post-operatively [17]. Creating a network of standardized collections of PROMs in all OECD countries will help investigate the determinants of quality in healthcare and to carry out national and international comparisons.

The IRCCS Rizzoli Orthopedic Institute (IOR) in Bologna, Italy, was selected as one of the pilot centers to launch the PaRIS initiative in Italy, with the aim to accelerate the adoption and reporting of validated, standardized, internationally comparable patient-reported indicators. Since 2000, local health authorities appointed IOR to implement and manage the Registry of Orthopedic Prosthetic Implants (RIPO), a region-wide registry which collects joint implant data from all public and private hospitals in the Emilia-Romagna Region in Italy, an area of 4.5 million inhabitants [18, 19]. In this specific geographic region the information related to patient reported functional outcomes after joint replacement have never been collected. Therefore, information on health-related quality of life in this specific population is not available.

This paper describes the protocol of a study (PaRIS-IOR) that aims to implement the collection of a set of PROMs within an existing implant registry in Italy. The study will also investigate the temporal trend of PROMs in relation to the type of prosthesis and the type of surgical intervention. This can be useful for clinicians, surgeons and policy-makers in order to improve healthcare quality in a specific geographic region.

## Methods

### Design and setting of the study

The PaRIS-IOR study is a prospective, single site, cohort study that will be conducted at the IOR, a third-level mono-specialty hospital in Bologna, Italy. About 60% patients admitted to IOR for joint replacement surgeries come from other regions or countries, therefore IOR population can be considered nationally representative. PROMs data will be linked to the Registry of Orthopedic Prosthetic Implants (RIPO). This registry collects on a regular basis data of patients undergoing hip, knee and shoulder replacement. Collected data include patients’ demographics, pathology leading to joint replacement, type of surgical procedures, and the characteristics of the implant.

PROMs questionnaires will be given to patients awaiting surgery by specifically trained researchers within 30 days before surgery for hip, knee or shoulder arthroplasty, and then at 6 and 12 months following surgery.

PROMs data will be merged with those routinely recorded in the RIPO registry database [20] and with hospital discharge records to determine the frequency of adverse events, implant failures and hospital re-admissions.

### Study population and recruitment procedures

The study population will include consecutive adult patients undergoing elective hip, knee or shoulder arthroplasty admitted at the 6 operative units that perform hip, knee and shoulder arthroplasty (Orthopedic and Traumatological Clinic 1, Orthopedic and Traumatological Clinic 2, Orthopedic and Traumatologic Clinic 3, Reconstructive Orthopedic Surgery - Innovative Techniques, Shoulder and Elbow Surgery, Prosthetic Surgery and revisions of hip and knee implants).

For elective hip, knee and shoulder surgery performed at the 6 operative units, the IOR has activated a Pre-Admission Plan (PAP). About 90% of patients who undergo elective surgery are included in the PAP, visited in a dedicated clinic within the hospital, where an orthopedic surgeon confirms the indication for joint replacement and an anesthesiologist assesses the patient’s eligibility to undergo surgery. From 15 to 30 days before the admission, patients are contacted by phone and notified about the admission date and the admission procedures. For these patients the recruitment will be carried out in a specific area during the pre-admission visit. Patients not included in the PAP (about 10%) will be enrolled directly at the operative units before the surgery.

Inclusion criteria are:

1. Males and females
2. Age 18–95 years
3. Elective primary hip, knee or shoulder arthroplasty
4. Willingness to be enrolled in the study, by signing the informed consents

Exclusion criteria are:

1. Severe cognitive impairment
2. Arthroplasty for musculoskeletal cancer
3. Patient not eligible for surgical procedures

### Study phases and intervention

**Fig. 1:**
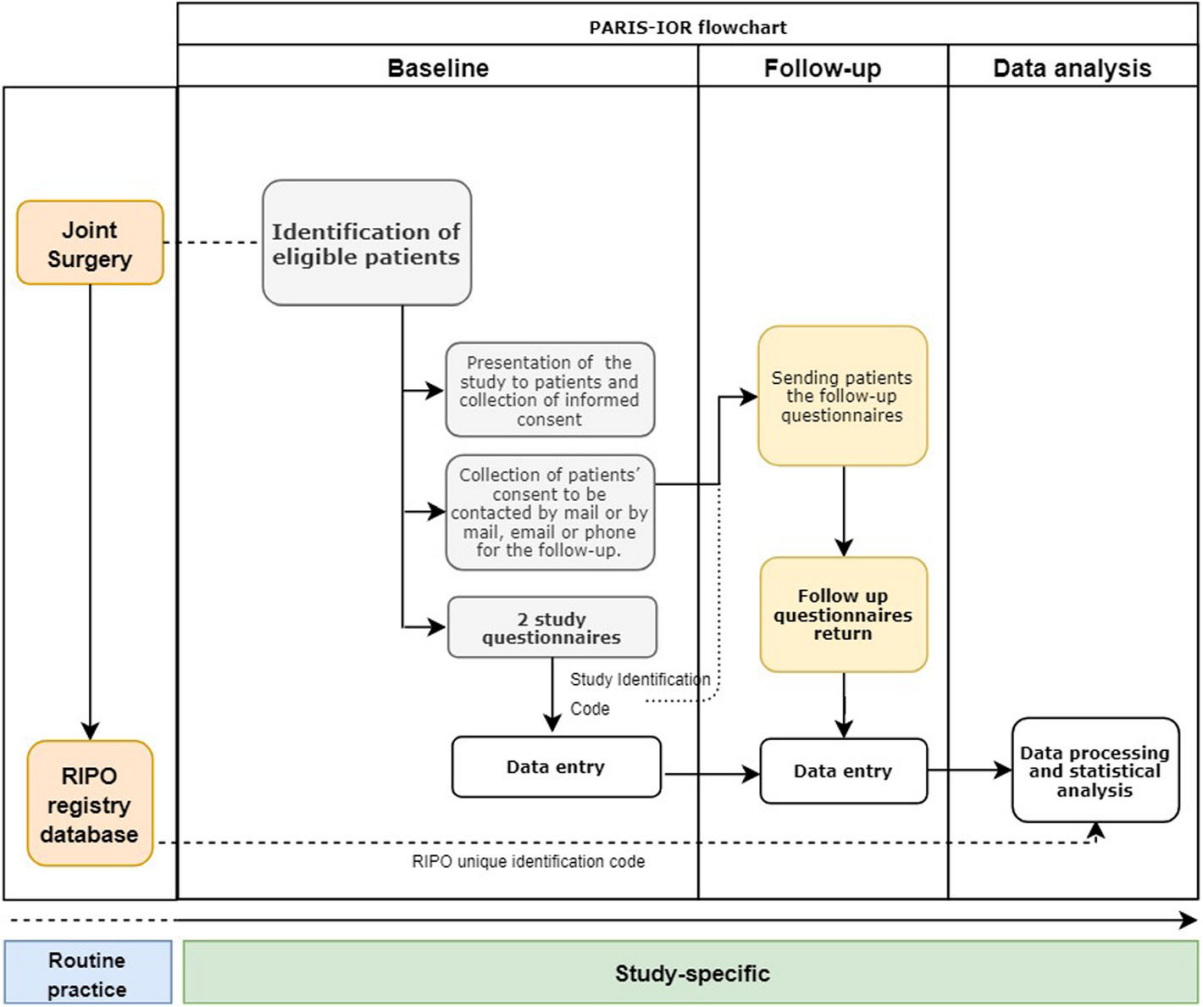
PaRIS-IOR flowchart including the pre-study routine practice for data collection in RIPO registry and the study phases (baseline, follow-up and data analysis)

The study consists of 3 phases (Fig. 1). At baseline, eligible patients that meet the inclusion criteria will be identified. The evaluation will be performed in the PAP clinic in the 15–30 days preceding the intervention or in the ward of the operative unit just before surgery; in this phase, patients who agree to participate to the study will be administered two self-report questionnaires (EQ-5D-3 L for all patients and the joint-specific questionnaire). The staff in charge of the distribution and collection of the questionnaires will follow a progressive number that will be the same used to input the data in the CRF and to match the patient record with the RIPO’s unique anonymized identification code.

The follow-up phase will be carried out at 6 and 12 months by mailing the same 2 questionnaires; participants will be asked to return the filled questionnaires. In case the questionnaires are not mailed back within 1 month, the participants will receive a reminder phone call.

Data analysis will be performed by the RIPO staff with the methodological support of the Biomedical and Health Services Research Unit of the University of Bologna.

### Instruments

Four validated questionnaires will be used in the study. One questionnaire (Euro Quality 5 Dimensions, EQ-5D-3 L) for the general assessment of the patient health status will be administered to all patients enrolled in the study [21]. According to the surgical procedure, patients will be administered with a joint-specific questionnaire: the Hip disability and Osteoarthritis Outcome Score – Physical function Short-form (HOOS-PS) for patients undergoing hip replacement [22]; the Knee injury and Osteoarthritis Outcome Score – Physical function Short-form (KOOS-PS) for patients undergoing knee replacement [23]; the American Shoulder and Elbow Surgeons Standardized Shoulder Assessment Form (ASES), for patients undergoing shoulder implants. This last questionnaire will be administered in a modified version that does not include the assessments performed by the orthopedic surgeon [24].

#### Euro quality 5 dimensions questionnaire (EQ-5D-3 L)

The Euro Quality 5 Dimensions EQ-5D is a standardized measure of health related quality of life used worldwide [25, 26]. It is a self-report instrument consisting of a descriptive system with five dimensions (mobility, self-care, usual activities, pain/discomfort, and anxiety/depression) and a visual analog scale (EQ-VAS) to measure overall health.

There are two versions of the instrument for use in the adult population, EQ-5D-3 L and EQ-5D-5 L. The version of EQ-5D used in this study (EQ-5D-3 L) uses three levels of severity (1 = no, 2 = some, 3 = extreme problems or unable to) in each dimension. We have decided to use the version with three response options (EQ-5D-3 L) rather than the version with five response options EQ-5D-5 L because it is shorter, faster and easier to administer in clinical settings where elderly people are treated. The EQ-5D health states, defined by the EQ-5D descriptive system, may be converted into a single summary score by applying weights to each of the levels in each dimension, as is typically done in cost utility analysis.

The visual analogue scale (EQ-VAS) will be used by participants to rate their health status between the worst conceivable health state (score = 0) to the best conceivable health state (score = 100).

The Italian version of EQ-5D has been validated by Savoia et al. [21].

#### Hip disability and osteoarthritis outcome score – physical function short-form (HOOS-PS)

The HOOS-PS is the 5-item the short-form version of the Hip disability and Osteoarthritis Outcome Score (HOOS), developed in English in 2003 [27, 28] to measure pain, symptoms, activity of daily living, sport and recreation function and hip-related quality of life from the patients’ perspective. The 5 items included in the short form measure physical function and are rated on five-point Likert scale (none, mild, moderate, severe, extreme), where 0 indicates no problems and 4 extreme problems. Item score can be transformed to a 0–100 scale with 0 indicating the worst problems and 100 indicating no problems The HOOS was translated and adapted to Italian, according to the international guidelines provided by Torre et al. [22].

#### Knee injury and osteoarthritis outcome score – physical function short-form (KOOS-PS)

The KOOS-PS is the 7-item short-form of Knee injury and Osteoarthritis Outcome Score developed in English by Roos et al. [29] to measure pain, symptoms, activity of daily living, sport and recreation function and knee-related quality of life from the patients’ perspective. Items are rated on a five-point Likert scale, where 0 indicates no problems and 4 extreme problems and normalized to a 0–100 scale, where 0 indicates extreme problems and 100 no problem. The Italian version of KOOS has been validated by Monticone et al. [23].

#### American shoulder and elbow surgeons standardized shoulder assessment form (ASES)

The American Shoulder and Elbow Surgeon Standardized Shoulder Assessment Form (ASES) was developed in 1994 for the assessment of patients with shoulder problems. It consists of a self-report section and a section including the results of physical examination filled out by the medical staff. The questionnaire used in the present study will be administered in a modified version that does not include the assessments performed by the orthopedic surgeon.

The ASES Subjective Form includes 11 items that measure pain (1 item) and function (10 items). The response to the pain question is rated on a 10-cm visual analog scale (VAS). The 10 items in the function area measure daily living activities.

The ASES also includes two general items: doing usual work and doing usual sport. These items are rated from 0 (unable to do) to 3 (not difficult). The final score is obtained by multiplying the pain score by 5 and the cumulative activity score by 5/3 and then adding the two scores. The total score of the questionnaire ranges from 0 (=worst outcome) to 100 (=best outcome). The ASES has been validated in Italian by Padua et al. [24].

### Endpoints

The outcomes of interest are functional and quality of life outcomes, measured through the total scores and the subscales of the administered questionnaires. For the joint-specific functional scales, we will use the total score; for the EQ-5D we will use the scores of the single dimensions, along with a VAS total score on a 0–100 scale to measure the overall health status. Other outcomes include adverse events, implant failures and hospital re-admissions at 30 and 90 days after discharge.

### Sample size

The study will include all patients undergoing elective hip, knee and shoulder arthroplasty in the 6 Operative Units, who will meet the inclusion criteria. We plan to enroll 1700 patients, of which 1100 with hip replacement, 500 with knee replacement and 100 with shoulder replacement in 2019, according to the number of arthroplasties performed at the IOR in 2016. We expect a 10% rejection rate and a 40% follow-up drop-out rate.

### Statistical analyses

Patients’ demographic and clinical variables (gender, age, body-mass index, comorbidity, clinical history, diagnosis) and surgical details (type of surgical procedure, surgical technique, antimicrobial prophylaxis, type of implant, acute and post-acute complications, and implant failures) will be extracted from RIPO. Adverse events and 30 and 90 days hospital readmissions will be extracted from the hospital discharge record database. For statistical analyses patients will be stratified according to the anatomic site of the surgical procedure (hip, knee and shoulder arthroplasty). Patients with a bilateral implant will be included in the analyses just once.

To analyze the temporal trend of functional and quality of life outcomes and to identify the factors influencing their trend, generalized estimation equations (GEE) and structural equation models (SEM) will be used. These techniques allow to obtain robust estimates even in presence of missing data. We estimated a 40% attrition rate and expected that non-compliance in returning the questionnaires is not linked to a systematic reason (for example, baseline level of joint impairment). However, it is possible that patients with specific characteristics (for instance very old patients or patients with less severe conditions) do not return the follow-up questionnaire because they are unable or not interested in providing information on their functional status. If there is a differential response to follow-up related to patients’ conditions, follow-up data may be biased. To determine the possible presence of this bias, sensitivity analyses will be conducted.

### Study duration and timeline

The study will have an overall duration of 30 months and will be considered in any case completed 6 months after the recruitment target is achieved.

The study timeline is the following:

- Months 1–12: Selection and enrollment of the patients and database implementation.
- Months 6–24: Follow up (6 and 12 months) and database implementation.
- Months 24–30: Analysis of data and final dissemination of results.

Due to the easy design and the low clinical and organizational impact of the study, the collection and analysis of PROMs are expected to continue and become structural in the RIPO registry beyond the end of the study. Within the OECD’s PaRIS Initiative it is planned to generate a considerable amount of internationally comparable PROMs data.

### Ethics and dissemination

This study has been approved by the AVEC research ethics committee board [30], ID PG0013646 on 11-29-2018. All patients will sign two written informed consents, one for privacy and one for participation. The protocol of the study is also available on the clinicaltrials.gov platform (ID NCT03790267). The study findings will be reported for publication in peer-reviewed journals.

### Financial burden

Considering the characteristics of the study, the costs are related to personnel (2 RIPO units for a total of 32,500 € for the study duration) and postal expenses (foreseen in 8000 € considering the number of patients to be enrolled in the study). These costs will be incurred within the RIPO and IOR funds, respectively.

## Discussion

This paper described the protocol of the PaRIS-IOR study. The study will integrate and combine PROMs related to patients undergone joint surgery with implant registry data and will contribute to investigate the temporal trend of PROMs in relation to the type of prosthesis and the type of surgical intervention. This will be useful for clinicians, surgeons and policy-makers to improve healthcare quality and the management of patients undergoing hip, knee and shoulder arthroplasty in a specific geographic region where information on health-related quality of life after elective joint replacement is unknown. The outcomes of highly common procedures such as joint replacement should be ideally collected within a specific country or a geographical region. In fact, differences in both pre-operative status and outcomes across international patient groups have been highlighted in previous studies. In a study of 12 European countries evaluating patients with advanced hip osteoarthritis, the level of pre-operative pain and disability have been reported to vary across countries [16] . Similarly, Lingard et al. [31] reported that patient from United Kingdom had significantly worse clinical outcomes one and 2 years after total knee arthroplasty respect to those from USA and Canada, after adjusting for confounding factors. Moreover, Gromov et al. [15] reported greater pain and poorer function in US than in the European patients. Thus, the creation of this national\regional registry-based PROMS collection would help to further clarify this controversy.

Moreover, the present study consists in measuring the functional and quality of life outcomes of the participants and combining them with the clinical, surgical and organizational data routinely recorded into the RIPO registry. There are conflicting results in the literature on which surgical and organizational factors are associated with an improvement in PROMs after joint surgery [11, 27, 28]. Prodinger and Taylor highlighted [9] the role of PROMs in realizing a bio-psycho-social perspective on health. On the other hand, the same authors reported that one of the main concerns for using PROMs in monitoring quality of healthcare is the lack of standards on what change in PROMs scores should be achieved when conducting a surgery for arthroplasty. Amlie et al. [11] found worse outcomes 1–3 years after primary total hip arthroplasty performed with the direct lateral approach rather than the anterior and posterolateral approaches, using PROMs data from 1476 patients. Conversely, Peters et al. found no relevant differences in PROMs improvements between the anterior and posterolateral approaches [10]. The PaRIS-IOR study will contribute to increase the consistency of scientific evidence in this area.

The PaRIS-IOR study, beyond providing specific information on interventions and outcomes, can help in building data collection standards potentially useful in comparison analyses. As proposed by OECD, it is important to have shared and standardized measures to make comparisons at the international level, but also to provide insights for clinical audit activities at a national, regional and single organization level. Healthcare professionals can improve patient selection and profiling for the most suitable surgical technique or type of prosthesis. This should be done in order to increase appropriateness, effectiveness, safety, and therefore quality.

The study has several strengths. First, the collection of PROMs within the longest running implant registry in Italy may provide important insights from a geographical region where systematic collections of PROMs do not exist yet. A second strength is the collection of data and PROMs related to shoulder arthroplasty. The high number of shoulder arthroplasties performed at the IOR (> 100 per year) allows extending the collection of functional and quality of life outcomes to this cohort of patients. Moreover, the design of the PaRIS-IOR study includes the collection of PROMs at 3 different time points: before surgery, at 6 and 12 months. This will increase the information related to patients’ reported outcomes, following them over a long period of time.

This study has two important limitations. First, the study will be conducted in one center, a third-level mono-specialty hospital. This implies the risk of selecting patients with a more severe case-mix. In the future, it is desirable for the study to be extended also to the first and second level centers where the remaining joint surgery interventions are carried out in the Emilia-Romagna region. Secondly, the collection of data will take place using paper forms for the collection of functional and the quality of life outcomes of the study participants. This is mainly due to the low compliance to the use of new technologies by the elderly patients who will be enrolled in the study. Instead, this should take place relying on the increasing use of digital application for the remote record of clinical and functional outcomes, including PROMs. We are evaluating options to digitally record PROMs data and integrate them automatically into each patient’s electronic medical record and then in the registry, overcoming the need for paper forms. From this point of view, however, the main problems relate to security, privacy and interoperability of the systems.

In summary, we believe this study could have important implications in targeting the factors influencing the evolution of functional and quality of life outcomes reported by patients after hip, knee and shoulder arthroplasty. Widening the view, the study will also represent the first systematic collection of PROMs related to arthroplasty in Italy and will contribute to increase the adoption of such important outcome measures to improve healthcare quality.

## Data Availability

Protocol and other information are available at: https://www.clinicaltrials.gov/ct2/show/NCT03790267

## Abbreviations

ASES: American Shoulder and Elbow Surgeon Standardized Shoulder Assessment Form
EQ-5D: Euro Quality 5 Dimensions
GEE: Generalized Estimation Equations
HOOS-PS: Hip disability and Osteoarthritis Outcome Score – Physical function Short-form
IOR: Rizzoli Orthopedic Institute
KOOS-PS: Knee injury and Osteoarthritis Outcome Score – Physical function Short-form
OECD: Organization for Economic Cooperation and Development
PAP: Pre-Admission Plan
PaRIS: Patient-Reported Indicator Survey
PROMs: Patient-Reported Outcome Measures
RIPO: Registry of Orthopedic Prosthetic Implants
SEM: Structural Equation Models
VAS: Visual Analog Scale
WHO: World Health Organization
WOMAC: Western Ontario and McMaster Universities Osteoarthritis Index
